# Supplementary material for: Biochar Mitigates the Negative Effects of Microplastics on Sugarcane Growth by Altering Soil Nutrients and Microbial Community Structure and Function
Source: Plants (Basel). 2023 Dec 27;13(1):83. doi: 10.3390/plants13010083 (PMC10781033; doi:10.3390/plants13010083)
Supplement: Supplementary file 1 [file plants-13-00083-s001.zip › plants-2777220-supplementary.pdf]

Supplementary tables

Table S1 The relative abundances of the most abundant phyla in different treatments.

	Taxon	Relative abundances (%) in different treatments					
		CK	Low PE	High PE	BC	Low PE+BC	High PE+BC
16S rRNA gene-based bacteria	Proteobacteria	40.44a	39.93a	42.35a	45.10a	38.27a	40.11a
	Chloroflexi	7.95a	9.49a	13.40a	8.28a	7.90a	7.70a
	Actinobacteriota	11.96a	16.29a	8.69a	8.95a	11.34a	13.44a
	Acidobacteriota	**7.71bc**	**8.78bc**	**7.36c**	**10.94a**	**9.35abc**	**9.81ab**
	Gemmatimonadota	**9.86a**	**7.62b**	**8.10b**	**8.20b**	**8.19b**	**5.88c**
	Verrucomicrobiota	5.09a	2.40a	3.40a	2.06a	2.48a	1.88a
	Bacteroidota	3.00a	2.56a	4.18a	5.31a	3.52a	5.84a
	Firmicutes	2.45a	2.17a	1.86a	2.39a	3.75a	2.89a
	Myxococcota	1.86a	2.21a	2.14a	1.88a	1.79a	1.96a
	Patescibacteria	0.24a	0.25a	0.50a	0.50a	0.24a	0.53a
phoD-harboring bacteria	Proteobacteria	**81.488ab**	**84.454a**	**83.605ab**	**79.338ab**	**85.482a**	**76.150b**
	Actinobacteria	11.616a	10.105a	8.268a	11.260a	6.226a	13.762a
	Planctomycetes	**0.008b**	**0.018ab**	**0.031a**	**0.027a**	**0.018ab**	**0.024ab**
	Acidobacteria	**0.005b**	**0.028a**	**0.019ab**	**0.018ab**	**0.010ab**	**0.011ab**
	Firmicutes	0.000a	0.004a	0.002a	0.002a	0.004a	0.000a
	Bacteroidetes	0.000a	0.000a	0.002a	0.000a	0.001a	0.000a
	Cyanobacteria	0.001a	0.000a	0.000a	0.000a	0.000a	0.000a

Different lowercase letters in the same row indicate significant differences among the six treatments (P<0.05).

Table S2 The relative abundances of the 10 most abundant genera in different treatments.

	Taxon	Relative abundances (%) in different treatments					
		CK	Low PE	High PE	BC	Low PE+BC	High PE+BC
16S rRNA gene-based bacteria	Pedosphaeraceae	**4.42a**	**1.64ab**	**1.99ab**	**1.56b**	**2.00ab**	**1.09b**
	Dongia	1.59a	2.16a	2.80a	2.96a	2.21a	2.45a
	TRA3-20	**3.86a**	**3.00ab**	**3.43a**	**3.20ab**	**2.33b**	**2.17b**
	SC-I-84	**2.97a**	**1.51ab**	**1.66ab**	**2.04ab**	**1.34b**	**0.70b**
	Subgroup_10	**1.58c**	**1.71bc**	**1.07c**	**3.27a**	**2.93ab**	**2.23ab**
	IMCC26256	0.65a	2.03a	2.18a	0.26a	0.64a	1.55a
	Sphingomonas	**2.78ab**	**3.66a**	**2.29b**	**2.78ab**	**2.66ab**	**2.54ab**
	Bacillus	**1.46ab**	**1.06ab**	**0.59b**	**0.64b**	**2.27a**	**1.17ab**
	Ellin6067	**2.46ab**	**1.90bc**	**2.71a**	**2.75a**	**1.40c**	**1.44c**
	Pseudomonas	0.68a	0.43a	0.98a	0.47a	0.81a	1.74a
phoD-harboring bacteria	Amycolatopsis	6.918a	1.606a	2.022a	5.005a	1.679a	2.417a
	Streptomyces	**0.560c**	**2.347a**	**1.461b**	**0.300c**	**0.543c**	**1.212bc**
	Bradyrhizobium	0.598a	0.265a	0.441a	1.884a	0.296a	0.237a
	Sinorhizobium	0.096a	0.161a	0.291a	0.156a	0.267a	0.078a
	Phaeobacter	0.056a	0.100a	0.050a	0.112a	0.110a	0.054a
	Pseudomonas	0.006 a	0.008a	0.018a	0.006a	0.007a	0.008a
	Actinoplanes	**0.048a**	**0.033ab**	**0.021ab**	**0.035ab**	**0.005b**	**0.037ab**
	Frankia	0.018a	0.018a	0.038a	0.013a	0.029a	0.017a
	Saccharopolyspora	**0.007ab**	**0.005ab**	**0.000b**	**0.013a**	**0.001ab**	**0.004ab**
	Stella	**0.007bc**	**0.015abc**	**0.018ab**	**0.019a**	**0.007bc**	**0.004c**

Different lowercase letters in the same row indicate significant differences among the six treatments ($P<0.05$).
